# Gut chemosensing: implications for disease pathogenesis

**DOI:** 10.12688/f1000research.9208.1

**Published:** 2016-09-30

**Authors:** Christopher J. Berg, Jonathan D. Kaunitz

**Affiliations:** 1 Medical Service, West Los Angeles Veterans Affairs Medical Center, Los Angeles, CA, USA; 2 Departments of Medicine and Surgery, UCLA, Los Angeles, CA, USA; 3 David Geffen School of Medicine at UCLA, Los Angeles, CA, USA

**Keywords:** Gut chemosensing, taste, TASR, Taste receptors, Artificial sweeteners

## Abstract

The ability of humans to sense chemical signals in ingested substances is implicit in the ability to detect the five basic tastes; sweet, sour, bitter, salty, and umami. Of these, sweet, bitter, and umami tastes are detected by lingual G-protein-coupled receptors (GPCRs). Recently, these receptors were also localized to the gut mucosa. In this review, we will emphasize recent advances in the understanding of the mechanisms and consequences of foregut luminal chemosensing, with special emphasis on cell surface GPCRs such as the sweet and proteinaceous taste receptors (TASRs), short- and long-chain fatty acid (FA) receptors, and bile acid receptors. The majority of these luminal chemosensors are expressed on enteroendocrine cells (EECs), which are specialized endocrine cells in the intestine and pancreas that release gut hormones with ligand activation. These gut hormones are responsible for a wide variety of physiologic and homeostatic mechanisms, including glycemic control, appetite stimulation and suppression, regulation of gastric emptying, and trophic effects on the intestinal epithelium. Released from the EECs, the gut peptides have paracrine, autocrine, and endocrine effects. Additionally, EECs have unique direct connections to the enteric nervous system enabling precise transmission of sensory data to and communication with the central nervous system. We will also describe how gut sensors are implicated in gut hormone release, followed by examples of how altered gut chemosensing has been implicated in pathological conditions such as metabolic diseases including diabetes and obesity, functional dyspepsia, helminthic infections, colitis, gastric bypass surgery, and gastric inflammation and cancer.

## Introduction to gut chemosensing

Chemosensors are proteins expressed on the cell surface that, when activated by small molecule ligands, generate neurohormonal responses that have profound effects on homeostatic mechanisms. In this review, we will emphasize recent advances in the understanding of the mechanisms and consequences of foregut luminal chemosensing, with special emphasis on cell surface G-protein-coupled receptors (GPCRs) such as the sweet and proteinaceous taste receptors (TASRs), short- and long-chain fatty acid (FA) receptors, and bile acid receptors. The functions of these GPCRs and identification of their respective ligands were elucidated by cloning, deorphanization, and subsequent molecular and functional characterization. The majority of these luminal chemosensors are expressed on enteroendocrine cells (EECs), which are specialized endocrine cells in the intestine and pancreas that release gut hormones with ligand activation. These gut hormones are responsible for a wide variety of physiologic and homeostatic mechanisms, including glycemic control, appetite stimulation and suppression, gastric emptying, and trophic effects on the intestinal epithelium. Released from the EECs, the gut peptides have paracrine, autocrine, and endocrine effects. Additionally, EECs have unique direct connections to the enteric nervous system in the form of neuropods, specialized cellular appendages that facilitate directed release of gut hormones onto enteric nervous cells, enabling precise transmission of sensory data to and communication with the central nervous system ^1, 2^.

We will also describe how gut sensors are implicated in gut hormone release, followed by examples of how altered gut chemosensing has been implicated in pathological conditions such as metabolic diseases including diabetes and obesity, functional dyspepsia, helminthic infections, colitis, gastric bypass surgery, and gastric inflammation and cancer. Not intended as an exhaustive review, our aim is to introduce the concept that the pathogenesis of diverse diseases and pathological conditions is linked by the common phenomenon of hormone release in response to the activation of specific receptors by luminal components.

## Chemosensors

### Taste receptors

***Overview.*** While the locus of taste reception has been attributed to the lingual sensors for centuries, recent studies have identified which sensors are activated by specific tastants. Of the five basic tastes (sweet, salty, bitter, sour, and savory [umami]), the sensations of sweet, umami, and bitter are conveyed by two GCPR families, whereas salty and sour tastes are sensed by ion-specific channels ^3^. A new and striking discovery is that some lingual TASRs, especially the GCPRs expressed on non-lingual tissues, including small bowel, liver, skeletal muscle, brain, and central nervous system, are involved in hormone release ^4, 5^. Within the gastrointestinal tract, two families of taste receptors (TAS1R and TAS2R) sense umami, sweet, and bitter tastes. These GPCRs are associated with the specific G-protein α-subunits α-gustducin and α-transducin, which mediate gustatory signal transduction pathways ^6^.

The first taste receptor family (TAS1R) is composed of three members—TAS1R1, TAS1R2, and TAS1R3—that form two heterodimers. The TAS1R1/TAS1R3 complex, known as the umami receptor, is a broad-spectrum receptor whose ligands include L-glutamate and other amino acids. Conversely, the TAS1R2/TAS1R3 complex, known as the sweet taste receptor, has broad specificity for sweet compounds, including carbohydrates, polyols, and non-nutritive sweeteners (NNS) ^7– 9^. The TAS2R receptor family, composed of some 25 different receptor subtypes specific to bitter-tasting molecules, is thought to have evolved for the detection of toxic substances ^8, 10^.

Both TAS1R1/TAS1R3 and TAS1R2/TAS1R3 are GPCRs coupled to a heterotrimeric G-subunit composed of α-gustducin and Gβγ subunits. Upon binding of the appropriate ligand, the activated G-subunit interacts with phospholipase C, releasing ATP via the inositol trisphosphate (IP _3_)/diacylglycerol (DAG)/Ca ^2+^ pathway ^7^. The subsequent increase in cytoplasmic Ca ^2+^ activates the cation channel TRPM5, which potentiates the effect of taste receptor activation ^9^. Within the gastrointestinal tract, the TAS1R2/TAS1R3 complex colocalizes with the gut peptide hormones glucagon-like peptide 1 (GLP-1), peptide YY (PYY), gastric inhibitory peptide (GIP), ghrelin, and cholecystokinin (CCK) in EECs, with subsequent gut peptide release following receptor activation ^8^. Similarly, sweet sensing by TAS1R2/TAS1R3 is important for the release of incretins GLP-1 and GIP and the expression of the glucose transporters SGLT-1 and GLUT-2 in the enterocytes ^4, 5^.

***Artificial sweeteners and TAS1R.*** NNS, which are high-affinity ligands for TAS1R2/TAS1R3 without caloric content, were previously thought to be a ‘healthy’ alternative to sugar. Nevertheless, by a variety of mechanisms, including the dissociation between sweetness and the caloric content, alteration of the gut microbiome, and interactions with intraluminal nutrient sensors such as the sweet taste receptors, NNS may cause previously unappreciated metabolic and hormonal alterations ^5, 11^. The influence of NNS on gut, exocrine, and endocrine organs through interaction with sweet taste receptors has been previously reported *in vitro* and in animal models, although the clinical implications have been controversial ^5, 12– 15^. Studies conducted in *in vitro* models have provided data to demonstrate that NNS dose-dependently release GLP-1, suggesting that NNS may contribute to the treatment of obesity or type 2 diabetes via incretin release ^14^. Nevertheless, the administration of NNS to animal models, particularly swine and mice, has effects similar to those of glucose such as increased expression of the intestinal sodium-coupled glucose transporter SGLT-1 with consequent increased glucose uptake ^16^. Furthermore, administration of the NNS erythritol and aspartame to diet-induced obese mice increased adiposity and insulin secretion, with no differences in food intake or weight gain, suggesting that activation of taste receptors by NNS affects energy utilization and lipolysis ^13^. In human subjects, administration of sucralose prior to a glucose bolus increases serum glucose and insulin concentrations, presumably through the activation of sweet taste receptors with subsequent increased expression of SGLT-1 and GLUT2 on intestinal epithelial cells and pancreatic β-cells, respectively ^12^. Extrapolation of these data may support the contribution of long-term ingestion of NNS with progressive insulin resistance and development of type 2 diabetes mellitus. Conversely, a recent small study of lean and obese individuals administered the natural polyol NNS xylitol and erythritol reported increased gut peptide secretion, particularly GLP-1 and CCK *in vivo*, along with prolonged gastric emptying ^15^. Interestingly, erythritol administration did not affect plasma insulin or glucose concentrations, whereas xylitol administration had a far smaller effect than did glucose. These results indicate that NNS have a spectrum of effect sizes, likely due to variant affinities for intraluminal chemosensors. Similar results have yet to be replicated in an *in vitro* study.

***Taste receptors in non-gastrointestinal tissue.*** Recently, there has been much interest in the expression of taste receptors in tissues and organs not typically associated with taste sensing. Taste receptors are expressed in pancreatic, liver, skeletal muscle, cardiac, and central nervous system cells ^4, 14, 17, 18^. New research has shown that muscle regulatory factors important for myogenesis and myoblast differentiation, specifically MyoD and myogenin, upregulate the expression of TAS1R3 in muscle cells ^17^. These new findings, along with the previously documented increased autophagy of skeletal muscle, liver, and related cells in TAS1R3 knockout mice, further suggest that muscle cell differentiation affects organ homeostasis through TAS1R3 expression alteration ^17^. One hypothesis explaining the increased observed autophagy in muscles cells in TAS1R3 knockout mice is that the sensors regulate the rate of autophagy in muscle cells during protein deprivation (e.g. starvation), as skeletal muscle is the largest depot of available amino acids ^17^. In another recent study, besides verifying that the TAS1R1/TAS1R3 complex is expressed on cardiac myocytes and fibroblasts, the authors also recognized that certain TAS2Rs have increased expression on cardiac myocytes in times of starvation, further supporting the extra-gustatory functions of taste receptors, especially with regard to metabolic regulation. Surprisingly, expression of the TAS1R1/R3 complex was not increased with starvation ^19^.

***Dietary modification and taste receptors.*** The effects of dietary modification on the expression of taste receptors and their associated G-protein subunits have been previously described for a variety of diets ^20, 21^. Changes in receptor expression may be related to diet-related alterations of the luminal content rather than to the diet itself ^20^. In a recent study, de Giorgio *et al*. reported a significant up-regulation of α-gustducin- and α-transducin-expressing cells in the foregut of pigs fed a short- and long-term high-protein diet, further supporting the growing body of evidence that luminal nutrient sensing by TASR complexes affects hormonal and metabolic regulation ^22^.

***Taste receptors and the immune response.*** Tuft cells (also known as brush cells) are another type of luminal sensory cell. Although long identified as being a type of gut chemosensing cell similar to umami and bitter taste receptors, their contribution to immunomodulation was only recently described ^23^. Recently, it was reported that the tuft cell population increased in mice that suffered from helminth infection. Upon further investigation, it was discovered that the tuft cells initiated a type II immune response against the parasitic infection via the cation channel TRPM5, likely from the activation of GCPR taste receptors ^24^. Through this study, it was determined that tuft cells are important for defense against intestinal parasitic infections, in that taste receptors, sensing parasites, release IL-25 which increases the rate of proliferation of tuft cells as well as initiates a type II immune response ^24^.

### Fatty acid sensors

***Overview.*** Similarly to the receptors that recognize sweet and savory molecules, there are several receptors expressed on EECs that detect intraluminal lipids belonging to a growing number of deorphanized GPCRs whose importance is currently being investigated and elucidated ^25^. The free FA receptors (FFARs) FFA1 (GPR40) and FFA4 (GPR120) sense long-chain FAs, whereas FFA2 (GPR43) and FFA3 (GPR41) detect short-chain FAs (SCFAs). FA ligands activate FFARs, releasing gut peptides such as CCK, GLP-1, and PYY, and also interact with FFARs expressed in the enteric nervous system ^26^. Also integral to the FA signal transduction pathway is the membrane-bound glycoprotein receptor CD36, which often colocalizes with other FA receptors. CD36 is hypothesized to recognize a wide variety of FAs at lower concentrations than do other FA receptors. Some have hypothesized that CD36 is necessary for the detection of physiologic concentrations of FAs, whereas other receptors, such as GPR120 downstream to CD36, are activated by higher concentrations of FAs ^27^. CD36 knockout models display decreased preference to fatty foods compared to their wild-type counterparts ^28^. Moreover, CD36 helps to mediate FA absorption in the duodenal mucosa ^29^.

Since the activation of FA receptors such as GPR40 expressed on pancreatic β-cells by specific agonists increases the rate of insulin secretion, GPR40 agonists have been investigated as a promising new therapeutic class for type 2 diabetes mellitus. A recent Japanese clinical trial reported similar reductions in hemoglobin A1c with the administration of TK-875, a synthetic GPR40 agonist, as compared with glimepiride (a sulfonylurea) with less risk of hypotension ^30^. Non-metabolizable analogues of gut peptides, particularly the incretins GLP-1 and GIP, are approved for use in the treatment of type 2 diabetes mellitus and obesity in the form of non-metabolizable GLP-1 receptor agonists and inhibitors of the principal peptide hydrolytic enzyme dipeptidyl peptidase (DPP)-IV ^31^, reflecting the emerging interest in the therapeutic potential of gut hormones ^9^. Also, synergistic improvement in glucose metabolism can be achieved by combining GPR40 agonists with DPP-IV inhibitors, which prolong the plasma half-life of GLP-1 ^32^.

***Luminal lipid sensors and functional dyspepsia.*** The luminal sensing of SCFAs, a major product of bacterial fermentation of indigestible fibers, has gained recent interest due to the nexus between small intestinal bacterial overgrowth and mucosal immune function. It was reported for the first time that the activation of duodenal luminal SCFA sensors increases the secretion of luminal bicarbonate via receptor-specific pathways ^26^. Activation of FFA1 and FFA3, whose agonists are LCFAs and SCFAs, respectively, increased the rate of mucosal bicarbonate secretion through a GLP-2-dependent pathway. Moreover, FFA2 increased the rate of bicarbonate release via 5-hyroxytryptamine (5-HT) and muscarinic neural pathways ^26^. Diseases like irritable bowel syndrome and functional dyspepsia are associated with small intestinal bacterial overgrowth, which significantly increases the amount of intraluminal SCFAs. Additionally, the functional dyspepsia symptoms of bloating and altered bowel habits improve with the administration of 5-HT antagonists, supporting the hypothesis that functional dyspepsia symptoms are directly related to the dysregulation of SCFA sensing and 5-HT release ^26^.

There have been exciting developments in the areas of SCFA sensing as it relates to colonic immune function. In a recent murine study, SCFA diet supplementation, and therefore an increased luminal concentration of SCFAs, was associated with an increase in colonic regulatory T cells and expression of the key anti-inflammatory cytokine IL-10 ^33^. Additionally, in mice with induced colitis, supplementation with SCFAs decreased the severity of colitis manifestations, an effect not observed in GPR43 knockout mice, suggesting that intraluminal detection of SCFAs is a key element of the connection between intestinal immune function and the gut microbiota.

***Duodenal expression of free fatty acid receptors and obesity.*** Recent studies have suggested that the rate of post-prandial gut peptide secretion is decreased in obesity, suggesting dysregulation of nutrient sensing and subsequent gut hormone release that affects energy intake in obesity ^29, 34^. A recent human study examining the difference in duodenal FA receptors among lean, overweight, and obese individuals noted that the density of FA receptors and EECs correlated with body mass index (BMI). Specifically, the authors reported increased expression of duodenal CD36 and GPR120 with decreased density of GLP-1- and CCK-containing EECs with increasing BMI. This study further suggests the importance of nutrient sensing in the development of metabolic derangements ^29^. Interestingly, a recent study indicated that high-fat-diet-induced obese mice had decreased CD36 expression on taste cells and decreased fat preference compared to mice fed standard chow ^27^. This discordance may suggest tissue-specific regulation of taste sensor expression or that alterations in nutrient sensing may contribute to the metabolic dysregulation associated with obesity.

### Bile acid sensors

***Overview.*** Bile acid receptors are non-nutrient chemosensors that help to mediate the release of the incretins GLP-1 and PYY, hormones that improve the response of EECs to glucose. The GPCR bile acid receptor TGR5 (GPBAR1) is expressed in a variety of tissues, including small intestine EECs, pancreatic β-cells, thyroid gland, brown adipose tissue, immune cells, and cardiomyocytes ^35^. Bile acid receptors contribute to bile acid homeostasis, glycemic control, and energy expenditure as well as to immunoregulation ^36^. Additionally, the metabolic improvements following bariatric bypass surgery (duodenal-jejunal bypass and Roux-en-Y gastric bypass) are hypothesized to be due in part to alterations of the composition and amount of bile acids that reach the distal small intestine, which are thought to interact with enteroendocrine L cells in an as-yet-unidentified manner and stimulate GLP-1 and PYY secretion ^7, 37^. Furthermore, post-surgical alterations of the luminal content are hypothesized to contribute to the magnitude of postoperative weight loss and improvements in glucose regulation via changes in gut hormone release. Candidate contributors to metabolic improvements include the incretins GLP-1 and PYY, leptin, and bile acid receptors ^37^. TGR5 has also been implicated in duodenal mucosal protection owing to the increased rate of duodenal bicarbonate secretion in response to luminal TGR5 ligands via a GLP-2 pathway ^38^. Additionally, TGR5 activation decreases the release of many pro-inflammatory cytokines in response to lipopolysaccharide (LPS) via inverse modulation of the NF-κB signaling pathway ^36^.

***TGR5 and gastric inflammation.*** *Helicobacter pylori,* a major cause of gastric and duodenal ulcers and a carcinogen associated with gastric adenocarcinoma and lymphoma, utilizes a type IV secretion system to initiate an inflammatory response via the delivery of the virulence factor cytotoxin-associated gene A ( *CagA*) ^39^. *CagA* activates the Ras-Raf-MEK-ERK kinase cascade, which serves to phosphorylate other protein kinases and gene-regulatory proteins such as NF-κB and activator protein-1 (AP-1). Activation of these transcription factors increases the release of the pro-inflammatory cytokines IL-8, CXC-chemokine ligand 2, and human β-defensin-2, among others ^39^. A recent murine study reported that TGR5 overexpression and subsequent ligand activation inhibits LPS-mediated gastric inflammation by blunting the pro-inflammatory response to the small molecules IP-10, TNF-α, and MCP-1 via inhibition of the NF-κB signaling pathway ^40^. These results suggest that TGR5 additionally protects against gastric inflammation and that TGR5 may be a potential therapeutic target for inflammatory and malignant gastric disease. Similarly, TGR5 knockout mice were more susceptible to LPS-induced gastritis, presumably due to the decreased inhibition of the NF-κB signaling pathway and thus greater inflammation. Moreover, activation of TGR5 significantly inhibits STAT3 phosphorylation and transcriptional activity in gastric cancer cells, which suggests that TGR5 is a potential tumor suppressor via suppression of the STAT3 pathway, a commonly constitutively active pathway in cancer cells ^41^.

## Conclusions

Luminal chemosensors are integral for the proper functioning of the gastrointestinal tract and are important for integrating gut and endocrine hormonal responses to changes in the luminal content and for communicating with the central and enteric nervous systems. Furthermore, these chemosensors are also implicated in altering nutrient palatability and sensitivity and in modulating inflammatory pathways, the dysregulation of which may contribute to diseases such as obesity, diabetes, and functional dyspepsia. This brief review highlighting some of the new advances in understanding luminal nutrient sensors underscores the need for continued investigations into the potential clinical and therapeutic applications of these receptors.
